# Use of mobile hospitals to improve access to health services and promote primary health care: lessons from Zambia (2011-2018)

**DOI:** 10.4314/ahs.v24i1.33

**Published:** 2024-03

**Authors:** Kabaso Kabwe

**Affiliations:** University of Johannesburg, Department of Politics and International Relations, Johannesburg

**Keywords:** Access to health care, mobile health, policy implementation, primary health care, Zambia

## Abstract

**Background:**

Mobile hospitals play a critical role in serving difficult to access populations. In 2011, they were introduced by the Zambian government to improve access to health care. However, little is known about and/or documented about their use in Zambia, and other similar settings where people rely on them to access critical health care, or have to travel long distances to the nearest health centre.

**Objective:**

To understand the use of mobile hospitals in Zambia and share lessons on their implementation that may be useful for similar settings. It describes their design, implementation, and challenges.

**Methods:**

The qualitative research employed document review, key informant interviews with 15 respondents, and observation of the operations of the mobile hospitals in the field.

**Results:**

The research finds that while they help to reduce inequities associated with accessing health services, there needs to be careful resource planning and addressing of the major issues in health care such as human resources, infrastructure, and disease prevention before long term use.

**Conclusion:**

The research not only highlights conditions that must be considered for the effective implementation of mobile hospitals, but also the need for engagement of various key stakeholders during agenda setting in order to build trust and buy in, which contribute to smoother implementation.

## Introduction

Since 1964 when Zambia gained independence, efforts have been made to address inequities in the health sector, including through the adoption of the primary health care approach in 1981 as articulated in the 1978 International Conference in Alma Ata^1^. Zambia has followed this approach through the health reforms which have been implemented since 1992. The vision of the reforms has been to “provide the people of Zambia with equity of access to cost-effective, quality health care as close to the family as possible^2^.” However, this is still a major challenge. Studies have shown that people with the greatest need for health services do not receive an equitable share as it is skewed in favour of the rich and people living in urban areas^3^.

The decision to introduce mobile hospitals to help address such inequities, in rural and peri-urban areas was announced in 2009^4^. This was met with fierce criticism from many sections of society. Many questioned the rationale for a mobile hospital that gobbles up millions of dollars for every outreach, as opposed to investing in health infrastructure^5^. Nonetheless, the prestigious mobile hospitals, purchased in 2010 for US$53 million through a loan from China became operational in 2011, providing various kinds of services to citizens^6^. There were nine mobile hospitals in total, one for each of the nine provinces at the time, operating on a schedule as determined by the relevant authorities in the respective province. From a population of about 17 million people, about 56 percent live in the rural areas while 54 percent live in the urban areas^7^. People in rural areas sometimes walk more than 20 kilometres to the nearest health centre, and even then, the health centre usually does not have sufficient skilled health professionals^8^.

Mobile health units are known for serving difficult to access sub-populations, with cost-effective preventive approaches to health care^9^. As providing suitable and timely services is one of the main challenges in the health care system of almost every country, mobile health services have been increasingly considered in health care policies in many countries in recent years to address public health issues in underserved segments of the population that are hard to reach by traditional systems^10^. Despite this and the advancement in technology and health, little is documented about mobile health units as a way of improving access to health care beyond crisis situations, and well-integrated into the health care system globally^10^. Compared to the typical mobile health unit which is more like a clinic focusing on preventive care and health education, the mobile hospitals offered a wider range of services, both preventive and curative.

In addition, there is limited research on the operations of the mobile hospitals in Zambia since implementation and little is documented about their use in terms of published articles. While the literature shows evidence in support of mobile health units in varying situations, most of it focuses on “small scale” operations of mobile clinics offering mainly primary health care, and sometimes run as projects by different organisations who usually receive funding from different partners. This was not the case here as the mobile hospitals were fully operated and funded by government, as an outreach service to rural and hard to reach areas. They also provided more than primary care, hence the term mobile hospital. There seems to be a lack of sufficient evidence which focuses on their use in providing secondary care, particularly in low resource countries.

The aim of this paper therefore is not to conduct an evaluation of the programme, or to investigate success or failure. Rather, it is to provide some insights into the design, operations and practical challenges of running such mobile hospitals in an under resourced health sector. It reviews and shares lessons on their implementation in Lusaka Province that may help to inform policy and practice not only in Zambia, but also in other similar countries, as improving health care delivery is high on the development agenda of most developing countries^11^. It is estimated that between 40 to 60 percent of people in developing countries live more than eight kms from a health facility^12^. In such cases, mobile health units could perhaps be useful, both to improve access, and the quality of health services offered to citizens.

### Delivery of health services using mobile health units

Mobile health units have been used in many settings to deliver health services. Examples of these contexts are neither exhaustive nor all-inclusive because some of these efforts are locally focused and may not be reported in the published literature. However, there are limited cases where they have been used the way that Zambia did, that is, going beyond provision of primary care, to include secondary care and being adopted as part of the package for delivering health care by the Ministry of Health.

A key feature of mobile health units is that they do not get stationed at a particular area but move from one location to the other according to their schedule. In many instances, they prove efficient in providing care and treatment in crisis situations like conflicts, natural disasters or emergency cases such as tsunamis, hurricanes or earthquakes. However, their use extends well beyond emergency relief as a variety of services are provided. These include promotive, preventative and curative depending on the population's needs^13^. Research has shown that mobile health units impact positively on access, cost and quality of health services by expanding access for vulnerable populations, improving chronic disease management and reducing costs for the clients^14^.

Typically however, in most developed countries mobile health units provide mainly routine emergency health services, preventive services such as immunizations, maternal health services like emergency contraception, blood pressure and blood sugar screenings, voluntary and counseling testing services and general advice on health care^15^. In developing countries, they are usually used to deliver general primary health care services or targeted services, to neglected or underserved communities where particular health services do not effectively reach the populations at risk. These include women, children and youths who are targeted for nutrition services, sexual and reproductive health services, and disease testing and screening activities such as for cancer, and infectious diseases such as HIV^15^,^16^. In Kenya for instance, a mobile diagnostic unit is implemented to increase access to imaging and laboratory services^17^ while in Malawi, mobile clinics are expanding access to health care in rural areas^18^. They help to remove logistical barriers such as transportation, finances, making appointments, long waiting times before appointments, and complex administrative processes.

### Zambia's health system and access to health services

Health care in Zambia is provided in five ways. The highest level is the third level hospitals, followed by second level hospitals, first level hospitals, health centres and lastly health posts. Health centres and health posts cover a limited geographical area, supported by the referral hospitals. Third level hospitals are thus the biggest and most specialized hospitals, while second level hospitals are mainly found at the provincial level, and first level hospitals at the district level^2^. Health management is done through the Ministry of Health headquarters at the centre, Provincial Health Offices and District Health Offices at the province and district levels respectively. The mission of the Zambian Ministry of Health is to ensure the provision of equity of access to cost effective quality health care as close to the family as possible. And although the population of the country is relatively small (about 17 million), it is geographically scattered, making delivery of equitable health services close to families a challenge^19^.

In terms of health financing, besides domestic funding from the government, donor support to the health sector is estimated at 56% of the total health expenditure^20^. However, it is mainly for vertical programmes such as HIV/AIDS, malaria and TB, which constitute a huge disease burden. There is also a growing burden of non-communicable diseases. Despite some improvements, the Ministry of Health unfortunately still operates under a critical shortage of staff. The rural areas have been the worst hit with staff population ratios almost 1:14,500 and 1:1,800 for doctors and nurses respectively, far below the WHO recommended staff population ratios of one health worker per four hundred people (1:400), with a health worker defined as a trained nurse, doctor, clinical officer, pharmacist, and laboratory, radiology and environmental technician^11^.

Major barriers to accessing health care include poor quality of health services, unavailability of medicines, inadequate staff, financial constraints, weak outreach programmes, and long distances to health centres^20^. Since most health workers are concentrated in urban areas, the few facilities in the rural areas lack both adequate and specialised equipment, and the staff to attend to them^21^. Abolishing user fees in 2006 to reduce financial constraints was the first step in trying to attain universal health coverage[22]. While access to health care and medicines is free in public health facilities where user fees were scrapped, the low stock of medicines mean that patients are sometimes simply issued with a prescription and have to buy their own medicine^23^. This is not feasible for poor households and limits their access to health care. Research has shown that a shortage of medicines is also a major factor in determining whether or not people will go to a health facility^8^. Another barrier to accessing health care is the physical accessibility of health facilities since the majority live in rural areas, and the distance to a health centre plays an important role in whether they are able to access health care or not. Because of increased costs related to transport, poor people especially struggle to access health care and medicines^23^. Mobile health service provision using mobile units has therefore been recommended in rural areas where people have difficulties with mobility, in order to reduce cost of access to health care^24^.

### Methodology

The approach to this research was qualitative, and the research design was a single case study of mobile hospital operations in Lusaka Province, Zambia. Data were collected through document review, semi-structured key informant interviews, and observation. Primary data were collected over a period of four months between August and November 2016. The mobile hospitals was observed in the field by the researcher to have a better understanding of operations. The mobile hospital observation during this period was done in one of the medium densely populated areas within Lusaka District where it was stationed for a period of seven days. An observation guide was developed, and notes were taken on different issues observed, including functional units of the mobile hospital, inflow and outflow of clients, general ambience, among others. To analyse this, data were categorized into meaningful categories according to the issues being observed.

Semi-structured key informant interviews were conducted with four main categories of respondents, carefully and purposefully selected based on their perceived knowledge of the topic. These categories were informants from the Ministry of Health headquarters; the first level hospital which co-ordinated the activities of the mobile hospital in the province; frontline health workers working in the mobile hospital; and lastly from some of the ministry's co-operating partners who play a key role in Zambia's health sector. The interviews aimed to explore themes related to agenda setting, design, and implementation. This included exploring key issues in the health sector, actors and process leading to the implementation of mobile hospitals, the operations of the mobile hospital in the province in terms of schedule, health cases attended to, challenges encountered, among other things.

A total of fifteen informants were interviewed and all of them were assured of complete anonymity. No demographic data about the informants was collected. Where the informants agreed, the interviews were recorded. Otherwise, the researcher also took notes. The interviews were conducted in English and transcribed verbatim personally by the researcher. The data wasere analysed manually using thematic analysis^25^. This involved identifying, analysing and reporting the emergent themes within the data, classified according to the above themes, and divided mainly between opportunities/positives and challenges/barriers

It is almost impossible to perform policy research without document review, one of the most frequently used methods in health policy research[2]^6^. Documents can be used in various ways throughout the research process, including during data collection, and analysis to help answer research questions^2^,^7^. Secondary data thus included reviewing various documents such as scholarly work, official documents and statements from the government of Zambia such as presidential speeches, and statutory and policy documents produced by the Ministry of Health, co-operating partners, and other stakeholders working in the health sector in Zambia, as well as print and electronic media reports. To review these documents, the READ approach^26^ was utilised, which is a systematic way of collecting documents and deriving data from them in the context of health policy studies at any level. The steps comprise: Readying the materials; Extracting data; Analysing data; and distilling the findings. Similar to the key informant interviews, the document review/analysis sought to explore issues related to the mobile hospital agenda, and implementation.

### Study setting

Lusaka Province is situated in the central part of Zambia and has the capital city, Lusaka within its boundaries. It is the smallest province in terms of surface area, second to the Copperbelt province in size and population. It has a population of about three million people, and has eight districts, including Lusaka District which is considered urban compared to the others. Mobile hospitals help to decongest referral hospitals and Lusaka has the largest referral hospitals, catering for patients from all over the country. Lusaka Province was also the first to launch mobile hospital operations in 2011 and hads therefore been implementing them longer than any other province. As such, it helps to study the implementation over a longer period of time.

### Ethical considerations

Ethical approval for this study was obtained from the Research Ethics Committee at Stellenbosch University (SU-HSD-002698), and from ERES Converge (Ref No. 2016-Aug-009), a research ethics committee in Zambia. Furthermore, authority was obtained from the Ministry of Health to interview staff and to observe operations of the mobile hospital in the field (MH/101/23/10/1). All participants voluntarily participated and gave written consent. Anonymity was assured and the right to freely withdraw or decline from answering any questions. In addition, permission was sought to record interviews, in which some participants declined. All recordings, transcripts and field notes are transcription were safely stored away stored in a password protected computer. The field notebook was also safely archived.

## Results

As the aim of this paper is mainly to share lessons from the implementation of mobile hospitals in Zambia, the results presented here thus explain the background behind the acquisition of mobile hospitals, their design, and the implementation process, including the benefits, and challenges in implementation.

### The mobile hospital agenda acquisition

The idea of mobile hospitals was introduced in 2009 by government. Suffice to say, up until implementation in 2011, as noted from both primary and secondary data, there was a lot of disapproval from various stakeholders including the donor community in the country who went as far as threatening to cut funding to the Ministry of Health^5^. They argued that it was not among the priorities, and that it would have been more beneficial in the long run if the government utilised the funds to instead improve the already existing health infrastructure. The opposition political parties accused government of being corrupt and wanting to benefit from the deal of purchasing the units because of their insistence to proceed with their purchase amid strong criticism^6^, and the seemingly exorbitant price at which the units were being bought given the poor status of the health sector. It was also seen as political move to appease the rural population as the general elections were approaching^6^.

Several informants stated that the idea of mobile hospitals was not initially supported because it was something that just sprang up, imposed by the authorities without proper consultation and without considering other options. *“If you want buy-in in a project, you do not go top-down.”* ([Interviewee G- Hospital staff] “*It was not a priority*”. [Interviewee H- Provincial hospital staff] *“There was need to engage stakeholders from the ground.”* [Interviewee K- Cooperating partner] “*There was so much secrecy and lack of information*.” [Interviewee N-Cooperating partner]. These were some of the statements expressed by interviewees. The Zambian government at the time, however, argued that the concept of mobile hospitals was not very different from that of the Zambian Flying Doctor Services; only that the latter moves in the air and the former moves on land but that they both target rural and disadvantaged areas^9^.

Implementation of the mobile hospital outreach only began in April 2011, a few months shy of the general elections that were held in September, the same year in which President Banda was seeking re-election. A senior informant from one of the Ministry of Health's partners explained in an interview:

*Health and politics cannot be separated, especially in a resource deprived country like ours. So, in a political campaign, you tell people something that will have an impact on them, and health is exactly that. To reap the benefits of school can take even up to 12 years until you finish your high school. But for health, today you have a headache tomorrow you don't. It's not a future investment, it's a now investment. And health has been used by all governments to campaign, past and present, and even future ones will do so. Health is one of the most politicised sectors in this country* [Interviewee M]

### The design of mobile hospitals

From the observation carried out in the field, a complete unit/hospital is a combination of seven large “vehicles” or “units” making up seven units/departments; an X-ray motor vehicle, a laboratory motor vehicle, a dispensary and an audio-visual motor vehicle, a mini theatre motor vehicle, an out-patient motor vehicle, a power and water supply motor vehicle, and a living motor vehicle which carried equipment plus a mini kitchen and other amenities for staff while on site. This was equivalent to a second level hospital as it had all the major departments required for such a hospital.

The power and water supply motor vehicle which contained a water tank, water purifier, water pump, generator set, and diesel tank was particularly useful in areas where power or water supplies are erratic. In addition to the above units, a tent transportation trolley for personal effects, food, tents, and other items was also part of the mobile health unit. A waste collection and transportation trolley collected and transported medical and human waste generated whilst on deployment.

### Mobile hospital implementation

Through the interviews and document review, the research established that the office responsible for outreach programmes at the Ministry of Health was expanded to strengthen the provision of health care through the mobile hospitals in 2010. This allowed it to ensure that the right staff was employed to oversee the operations. The Ministry ensured adequate training of medical staff to operate the units. Further, the coordination office at the provincial hospital mobilised the required staff with the right skills from wherever they may be within and outside the province, and from public and private sectors for the purpose of a particular outreach.

Following the change of government after the election of a new president in 2016, and therefore a new Minister of Health, changes were made in the ministry. Some departments were abolished, others merged, and other issues took centre stage. The National Health Strategic Plan (NHSP) (2017-2021) stated a recommitment to primary health care emphasising health promotion, disease prevention, and curative and rehabilitative services in close-to-client settings^2^,^8^. In this vein, the department responsible for the mobile hospitals was abolished and their management was placed under another department. This affected the budget as the mobile hospital operations were no longer considered a separate activity with their own budget, rather, a complementary service delivery mode to hard-to-reach areas. A key informant interviewee at the Ministry of Health revealed that this posed a challenge in terms of fund allocation as it was dependant on how the specific directorate allocated funds. He commented:

*In the near future when the units wear out, there will be no need for their replacement as they would have to be done away with due to the huge costs involved, both financial and human resources. Things have already started changing going by the policy decisions that had been made so far.”* [Interviewee B].

In addition, interviews with health workers revealed that the mobile hospital was scheduled to go for outreach at least once a month, for a period of between ten and fourteen days on average in a particular district. Sometimes, the mobile hospitals would also operate in urban districts areas like Lusaka district to help decongest the health facilities, especially in peri urban or densely populated areas. Before an outreach, the provincial hospital coordinated with the District Medical Office in the respective district to determine the kinds of medical cases to be anticipated in order to get the right expertise. The staff of the mobile hospitals were therefore drawn from across the province, depending on which health facility had the expertise required for the particular outreach. However, it was revealed in some interviews that a lack of adequate human resources was a major drawback. The fact that outreach programmes depended on skilled staff such as surgeons and theatre nurses who were mainly employed at tertiary hospitals meant that these facilities were deprived of critical staff when the mobile hospital was out in the field.

The local health facility in the respective districts also sent word around to alert the community of the date when the mobile hospital would be in the area. Districts close together would often be visited in one outreach if the mobile unit finished earlier than expected in another district. The service point for the unit was determined as one that had access points to a levelled ground, water, electricity and sanitary facilities amongst other fixtures^2^,^9^. Schools or churches usually met those requirements. If someone had a serious ailment that could not be adequately handled while the team was in the field, they were referred to a higher hospital, usually the district or provincial hospital.

However, interviews revealed that it was very expensive to run the unit and that a minimum of about US $8,500 was required for an outreach of about ten days. Largely due to funding constraints, the mobile unit was usually in the field for less than ten days, when the ideal was between ten and fourteen days^27^. Operational costs were listed as the main challenge in the implementing mobile hospitals. Besides logistical issues, the equipment was also expensive to maintain. Indeed, at the time of collecting this data, the X-ray machine had broken down and was awaiting repairs. As a result, whenever the unit was going for outreach, staff carried the machine from the provincial hospital. This meant that the hospital remained deprived until the mobile hospital returned. The National Health Strategic Plan (2017-2021) also acknowledges that the constant breakdown of equipment in the mobile hospitals, particularly laboratory and X-ray equipment was a key constraint in implementation^26^.

### Health care services and benefits

Various types of mobile health clinics offering various services have been piloted before in different parts of Zambia, either run or supported by charity organisations, and targeting rural populations for campaigns such as immunisation, antenatal and other primary health services^30^. In most cases, they are set up by charities in parallel to the public health system^7^,^18^, ^3^. In this case however, they were integrated into the national health system and implemented by the Ministry of Health. The mobile hospital provided a wide range of services free of charge to all as contained in the Basic Health Care Package^2^ as well as those offered at any first or second level health facility. It was envisaged that about nine million people would benefit from affordable, cost-effective and quality health services through this mode of delivery.^29^

A key informant from the Ministry of Health explained “there is a gap in the provision of health services. People in urban areas have access to all these higher levels of health care while the ones in rural areas access the lower levels of care, so the mobile hospital takes second level health care to these places (Interviewee A). The services offered included medical and paediatric, surgical, laboratory, radiological, pharmaceutical, physiotherapy, oral and eye health, rehabilitation and maternal and child health services, and cancer screenings, either on an out-patient or in-patient basis.

However, surgical cases were attended to more than any other cases. The presence of the mobile hospital in an area where such services were lacking was considered a great opportunity for the community to have their longstanding health issues attended to. In 2015 for instance, over 400 surgical operations were performed in the province^21^. The feedback received from health workers and informants from the Ministry of Health on the utilisation of the services was positive. They expressed satisfaction with the services and viewed the hospitals as a good initiative that was offering free services, having witnessed first-hand how people benefited, especially in rural and peri-urban areas.

### Challenges

Despite the benefits of the mobile hospitals to the users, interviews revealed that it was very expensive to run the unit and that a minimum of about US $8,500 was required for an outreach of about ten days. Largely due to funding constraints, the mobile unit was usually in the field for less than ten days, when the ideal was supposed to be between ten and fourteen days^2^,^9^. Operational costs were listed as the main challenge in running the mobile hospitals. Besides logistical issues, the equipment was also expensive to maintain. Indeed, at the time of collecting this data, the X-ray machine had broken down and was awaiting repairs. As a result, whenever the unit was going for outreach, staff relied on the X-ray machine from the provincial hospital, which had to be carried to the field. This meant that the hospital remained deprived until the mobile hospital outreach was completed for the period. The NHSP (2017-2021) also acknowledges that the constant breakdown of equipment in the mobile hospitals, particularly laboratory and X-ray equipment was a key constraint in implementation^2^,^8^.

## Discussion

The aim of universal health coverage is to ensure that all people, regardless of where they live, have a fundamental right to accessing adequate and reliable health care. Aiming for this through the use of the mobile hospitals, a number of things worked well and others did not. The research showed that the elections provided a window of opportunity for their implementation, arguably as a way for the governing party to campaign for the rural vote. The promise of better health is capable of attracting voters and has often been used to campaign in elections, placing health at the top of the developmental agenda. Nonetheless, the provision of free health care was helpful in removing some of the barriers associated with accessing health care services not only for people living in rural areas, but also those in urban areas. It encouraged the community members to come out in large numbers to access the various services, as most people are unable to pay out of pocket to seek specialised care.

In addition, the integration of this outreach service within the health system meant that government provided all the funding and support, including medicines and staff payments. This was good for coordination and prioritisation of cases as there were no restrictions as to what cases would be dealt with, unlike the case with vertical funds. The provincial hospital being the base for the mobile hospital to determine the schedule for outreach to all districts also contributed to capacity building among health workers, and to the provision of quality and cost-effective services as the experts from around the country were sent to areas that for the most part, did not have these services.

However, some aspects were challenging. While the general concept may have been good, it appeared that the Ministry of Health was not ready to sustain such a programme. By 2017, significant policy changes had been made in the administration and management of affairs in the ministry which affected resource allocation to the mobile hospital operations. The lack of resources and the critical shortage of staff affected operations as the mobile hospital did not have fixed staff, thus relied upon skilled staff from different facilities. Maintaining the equipment also proved to be a costly undertaking, and all in all resources became few and far between. This was also in part due to a lack of support from partners, as government eventually could not sustain the programme on its own. It is worth noting that cooperating partners play a huge role in the Zambian health sector, offering both technical and financial support for various programmes. The mobile hospital programme was implemented from 2011 until 2018 when it was discontinued owing partly to financial constraints. Therefore, there was only a window of when they were operational.

The corruption allegations did not instil public confidence in the government to procure and operate the units. Even though this perception did not affect uptake of services among community members as clearly the need was there, there was a lack of support and confidence from co-operating partners for this initiative, resulting in government having to exclusively fund it which proved very expensive and unsustainable in the long run. For a country like Zambia which is heavily reliant on donor funds, getting buy in from partners is helps to ensure success of programs. Thus, the dialogues during the policy discussions before implementation could have been more open to all those with divergent views.

Being the underlying strategy that the Ministry of Health follows for delivering health services, primary health care was neglected in the operations of the mobile hospital as the focus was mainly on surgical cases. This meant that the goals of health promotion and disease prevention were not being met adequately, which further had implications for receiving donor funding to support the programme. While this afforded the community a chance to have a specialist examine and treat their case, it was also difficult to have them return for review as this would entail referral usually to a district or provincial hospital, which brought its own logistical challenges. A focus on primary health care from the onset would have perhaps been more sustainable and in line with the NHSP (2017-2021) which placed primary health care as the main vehicle of health service delivery during the five-year implementation period.

Mobile hospitals were introduced on the premise that they would operate mostly in rural and peri-urban areas. However, that was not the case as they were also operating in urban areas like Lusaka district. Because health facilities are usually overwhelmed by large numbers against few staff, the use of the mobile units in the district was meant to help decongest and minimise pressure on these facilities. However, the problem with having the mobile hospital in urban areas where people had fairly good access to health services is that at the end of the day, the rural areas still remained deprived. The need to build more health facilities across the country and to staff them with the trained health workers therefore cannot be over-emphasised.

## Conclusion

By traveling to communities and offering free services, the mobile hospital helped to remove logistical barriers associated with accessing health care such as transportation, finances, making appointments, long waiting times before appointments are due, and complex administrative processes. The study however shows that this type of mobile health units- the mobile hospital that was offering more than primary care were expensive to run in a low resource setting such as Zambia, and where shifts in policy easily affect implementation. They should therefore be carefully analysed before implementation anywhere. The findings highlight the importance of planning interventions that target disease prevention and are aimed at individuals. It is also a missed opportunity to have them operate in this crucial time of the COVID-19 pandemic to overcome some of these barriers, particularly as health facilities are reported to be overwhelmed. While the mobile hospitals had the potential to contribute to improving health outcomes, major pressing issues in the health sector needed to be addressed to fully harness their benefits as they are not equipped to provide a continuous service compared to fixed health facilities. This confirms that they cannot be used as a substitute for static facilities even in under resourced communities, rather they can be used to provide adhoc services.
